# Lifestyle behaviors and stress are risk factors for overweight and obesity in healthcare workers: a cross-sectional survey

**DOI:** 10.1186/s12889-023-16673-w

**Published:** 2023-09-14

**Authors:** Xinyue Guo, Shaoqing Gong, Ying Chen, Xiaohui Hou, Tong Sun, Jianqiang Wen, Zhiyao Wang, Jingyang He, Xuezhu Sun, Sufang Wang, Xue Feng, Xiangyang Tian

**Affiliations:** 1 Department of Nutrition and Food Hygiene, School of Public Health, Anhui Medical University, Anhui, 230031 China; 2 Luohe Medical college, Henan, 462002 China; 3 Chinese Center for Health Education, Beijing, 100011 China; 4 Shandong Center for Disease Control and Prevention, Shandong, 250013 China; 5 Gansu Province Traditional Chinese Medicine Development Center, Gansu, 741021 China; 6 Health Promotion and Education Center of Xinjiang Uygur Autonomous Region, Xinjiang Uygur Autonomous Region, 843199, Xinjang, China; 7 Health Education Institute of Henan Center for Disease Control, Henan, 450004 China; 8 Center for Lifestyle Medicine, Fuwai Hospital, Chinese Academy of Medical Sciences, Beijing, 100037 China

**Keywords:** Healthcare workers, Overweight/obesity, Risk factors, Stress

## Abstract

**Background:**

Overweight and obesity have become major public health concerns worldwide. Persistent stress can activate the human hypothalamic‒pituitary‒adrenal axis (HPA) and increase the intake of “self-rewarding food”, thereby raising the incidence of obesity. Health care workers (HCWs) experience higher workloads and mental stress than workers in many other industries, which may put them at increased risk for overweight/obesity. However, few studies have been carried out on overweight and obesity among HCWs in China, and the overall scenario and behind-the-scenes factors of their overweight and obesity are unknown. The aim of this study is to understand the epidemic of overweight and obesity and risk factors among Chinese HCWs.

**Methods:**

Based on a cross-sectional web survey design, 23,234 HCWs from 100 health institutions in 5 provinces/autonomous regions/municipalities across China were sampled to answer a self-administered questionnaire that was purposely developed using a multi-staged clustered random-sampling method. Chi-square test and ANOVA were performed to compare variables between two or more groups. Univariate analyses were conducted to identify the influence of self-reported persistent stress and/or recurrent anxiety/depressed mood on lifestyle behaviors. A multivariate binary logistic regression model was used to analyse the risk factors of overweight/obesity.

**Results:**

Among the respondents, 34.26% were overweight, and 11.22% were obese. Most of the respondents had regular exercise habits (68.17%), had habitually stayed-up late (65.06%) and had been affected by persistent stress and/or recurrent anxiety/depressed mood (62.04%). A higher proportion of those with persistent stress and/or recurrent anxiety/depressed mood than those without habitually staying-up late (76.18%); consumed take-out food (54.92%), fried food (49.93%), snacks or desserts (50.51%); drank sugary drinks (46.57%); smoked (14.27%); and drank alcohol (23.34%). Gender (Female) (OR: 0.314, 95%CI: 0.292–0.336), age (OR: 1.742–2.334, 95%CI: 1.544–2.858), education (OR: 0.620–0.728, 95%CI: 0.445–0.973), living and working area (OR: 1.271, 95%CI: 1.192–1.355), breakfast (OR: 0.898, 95%CI: 0.839–0.960), fried food (OR: 1.133, 95%CI: 1.048–1.224), and alcohol consumption (OR: 1.111, 95%CI: 1.017–1.214) were factors for overweight/obesity. All of the aforementioned results were significant (*P* < 0.05).

**Conclusions:**

The overweight/obesity rate of Chinese HCWs is rather high, which might be directly associated with lifestyle behaviors. However, these behaviors fundamentally originated from persistent stress and/or recurrent anxiety/depression, mediated by lifestyle behaviors. Substantial measures should be taken for stress reduction and mental health promotion for overweight/obesity prevention and control among HCWs.

## Background

Overweight and obesity have become a major public health concern worldwide. In 2015, the global prevalence of obesity was 10.8% in adult males and 14.9% in adult females [1]. Overweight and obesity increase the risk of hypertension, coronary heart diseases, certain cancers and other chronic diseases [2]. Between 1990 and 2017, the number of deaths and disability-adjusted life years (DALYs) attributable to high body mass index (BMI) doubled globally [3].

Overweight and obesity can have a direct impact on daily activities and quality of life and even lead to mental and emotional disorders, [4] damage to work ability, sickness absence and presenteeism, [5] and may also increase occupational injuries. One study showed that overweight and obese employees had higher work costs from absenteeism and loss of normal workday productivity due to medical visits, insurance and disability losses than normal-weight employees [6]. Another study also indicated that obesity could even increase the risk of COVID-19 infection [7].

Overweight and obesity are traditionally believed to be caused by caloric intake greater than caloric expenditure [8]. The known risk factors for overweight and obesity include unhealthy habits, education, socioeconomic status, unhealthy diet and lack of regular exercise [9]. However, the latest research revealed that fat accumulation in the body is associated with human neuroendocrine activity. Research shows that persistent stress could activate the human hypothalamic‒pituitary‒adrenal axis (HPA), boost the secretion of cortisol, affect sleep, and increase the intake of “ self-rewarding food” (high fat and high sugar), thereby raise the incidence of obesity, especially abdominal obesity [10]. Furthermore, stress management disorders can lead to negative emotional problems such as anxiety and depressed mood, resulting in increased appetite and an increased risk of overweight and obesity [11, 12].

Healthcare workers (HCWs) are considered to experience a heavier workload and mental stress than their counterparts in many other industries due to their longer working hours, less personal time and higher burnout rate, possibly making them more susceptible to the risk of overweight/obesity [13]. Over the past three years, the extra burden from the COVID-19 pandemic has been substantial for healthcare personnel. A systematic review and meta-analysis of anxiety-related symptoms among healthcare personnel affected by the pandemic reported that 42% showed characteristics of anxiety, 40% acute stress, 37% burnout and 32% PTSD, and among emotional symptoms, depression accounted for 33% of sleep disorders while insomnia explained 42% of those [14].

Studies conducted in some healthcare institutions found that the overweight and obesity rates among HCWs were 70.7% in South Africa, [15] 45.9% in Brazil, [16] 40.9% in Germany, [17] 35.0% in Italy [18], and 69.1% in Britain [19]. By the end of 2020, the total number of HCWs in China reached almost 13.5 million, [20] but there have been few studies on the overweight and obesity rate in that population, and those studies were only based on the analyses of physical examination data of HCWs in individual medical institutions [21]. The overall scenario and behind-the-scenes factors of overweight and obesity among HCWs in China are unknown up to now.

This study aimed to investigate the prevalence of overweight and obesity and the fundamental associated risk factors among Chinese HCWs as well as the association between persistent stress and/or recurrent anxiety/depressed mood and overweight/obesity. This survey was performed during the COVID-19 pandemic from July to August 2022 by using a cross-China random sampling method. We seek to provide evidence for overweight/obesity intervention strategies and policy development among Chinese HCWs, and highlight priorities for future research.

## Methods

### Study design

Taking all Chinese HCWs as the study population, this study is based on a cross-sectional random-sample online survey using a structured self-administered questionnaire developed by the research group. The respondents’ relevant data were collected, including socio-demographics, health status, height and weight, persistent stress and/or recurrent anxiety/depressed mood, and lifestyle behaviors.

### Sampling strategy and eligibility criteria

A multi-staged clustered random sampling method was employed. First, 5 provincial administrative regions were selected at random from 32 provinces/autonomous regions/municipalities of China. Second, considering the inter-provincial difference and intra-provincial homogeneity, 20 healthcare institutions from each of the 5 provincial administrative regions were selected using a random systematic sampling method. Third, all HCWs of the selected institutions were surveyed as respondents. In total, 100 healthcare institutions were sampled.

The inclusion criteria of subjects were as follows: (1) age ≥ 24 years; (2) working in present healthcare institutions for at least 3 years from 2019 to 2022; (3) possessing and using a smartphone; and (4) providing informed consent and voluntarily participating in the survey.

### Survey tools

The survey questionnaire was developed by the study group. First, we conducted item pooling based on literature research and expert interviews. Second, a nominal group discussion with 10 senior experts in lifestyle/mental health was organized, and items (questions) with a face validity ≥ 0.8 were included. Third, we invited 10 HCWs to conduct a pre-test using the initial questionnaire to validate all the items. The weak items were removed or revised.

The final questionnaire comprises modules: informed consent, answering guide, socio-demographics, health status (Self-rated in the past three years (improving/worsening)), chronic diseases (with diagnosed hypertension, cardiovascular and cerebrovascular diseases, cancer, diabetes, COPD, osteoporosis, etc. [22] (Yes/No)) and control in the three past years (improving/worsening), height (m) and weight (kg) (based on latest annual physical examination records), and lifestyle behaviors (eating behaviors, regularly exercising, habitually staying up late (not falling asleep after midnight due to specific activities, voluntarily or involuntarily [23]. (Yes/No)), smoking (Yes/No) and alcohol consumption (Yes/No)). The socio-demographic variables included gender, age, education, and working and living locations (urban/rural). This study determined overweight and obesity based on BMI values (BMI = (Weight (kg))/(Height (m))^2^, and overweight was defined as 24.0 kg/m^2^ ≤ BMI < 28.0 kg/m^2^, and obese as BMI ≥ 28.0 kg/m^2^ [24].

To measure persistent stress, we used the following question: “During the past 3 years, have you frequently experienced feelings of being under too much mental or emotional pressure that made you angry/irritated/moody/frustrated, worried, or unable to sleep for at least 6 months?” Recurrent anxiety was measured by the following question: “During the past 3 years, have you experienced recurrent feelings of being nervous or restless or a sense of impending danger, panic or doom with increased heart rate/sweating/ accelerated breathing /trembling for at least 6 months?” To measure depression, we used the following question: “During the past 3 years, have you experienced persistent feelings of sadness, tearfulness, emptiness or hopelessness, and loss of interest or pleasure in most or all normal activities, such as sex, hobbies or sports, with angry outbursts, irritability or frustration, even over small matters for at least 6 months?” We collected data on eating and lifestyle behaviors such as consumption of take-out food (“Often”: 4–6 times/week), vegetables and fruits (“Regularly”: ≥ 4–6 times/week), breakfast (“Regularly”: 3–7 times/week), fried food (“Often”: ≥ 4–6 times/week), snacks or desserts (“Often”: ≥ 4–6 times/week), sugary drinks (“Regularly”: 4–6 times/week) and exercising (“Regularly”: ≥ 150 min/week), staying up late (“Habitually”: ≥ 4 times/week), smoking (Continuously or cumulatively for 6 months or more during their lifetime [25]), and alcohol drinking (At least once a week in the past year, including liquor, wine, beer, rice wine, etc. [26]).

The Cronbach's α of the final questionnaire was 0.82, with three factors explaining 63.55% of the total variance between the items based on exploratory factorial analysis (EFA).

### Data collection

The Wenjuanxing^Ⓡ^ Web Survey System [27] was used to generate a QR code of the electronic questionnaire. The investigators from each province contacted the liaison of each selected healthcare institution and sent the QR code to all the HCWs in the institution. The respondents were required to scan or press the QR code through WeChat to fill out the electronic questionnaire and submit it to the Wenjuanxing^Ⓡ^ backstage after completion. The WeChat ID was used to ensure that each respondent could only submit the questionnaire once. Every participant could obtain some daily necessities as a reward upon submission, such as napkins, towels, soap, hand sanitizer, etc..

Participants’ responses were anonymous and confidential and declared at the beginning of the electronic questionnaire. The respondents were also informed that data would be used for research purposes only. Participants completed the questionnaire directly connected to the Wenjuanxing^Ⓡ^ platform. Each submitted questionnaire was reviewed, and questionnaires with incomplete/missing items, incorrect completion, and obvious logical errors were regarded as invalid.

### Statistical analysis

Statistical analysis was performed using SPSS 25.0 software. The socio-demographic characteristics were analysed using descriptive statistics, and the quantitative data were represented by $\overline{\mathrm x}$ ± s. The chi-square test was used to assess the association between categorical variables and trends within a categorical variable. Analyses of variance (ANOVA) were performed to compare continuous variables between two or more groups. Univariate analyses were used to assess the influence of self-reporteded persistent stress and/or recurrent anxiety/depressed mood on lifestyle behaviors. The forced introduction method was used for binary logistic analyses, with overweight and obesity as dependent variables and socio-demographic characteristics and lifestyle behaviors as independent variables. *P* < 0.05 was considered to indicate statistical significance.

### Ethical declarations

This study was conducted in compliance with the Declaration of Helsinki. The Ethics Committee of the Chinese Academy of Medical Sciences Fuwai Hospital reviewed and approved the study procedures (No: 2021–1559). Informed consent of participants was obtained in accordance with ethical guidelines. This study abided by the principles of scientific research ethics, clearly stated the purpose of this survey in the preface of the electronic questionnaire, and strictly protected the privacy of all respondents. All respondents were required to be honest in their responses. The respondents were able to stop answering and leave the questionnaire at any stage before the end of the process, with no answers being saved. Respondents’ answers were saved by clicking on the “submit” button provided. Upon completing the survey, participants acknowledged their voluntary consent to participate in this anonymous study.

## Results

### Socio-demographic characteristics

A total of 23,234 HCWs (male: 31.80%, female: 68.20%) completed the questionnaire. The respondents’ average age was 39.85 ± 9.41 (30.44–49.26) years (ranging from 24 to 80 years), and more than 60% were among the 34–43 and 44–53 age groups. The proportion of males aged 44 years or older was higher than that of females (53.66% > 28.93%) (*P* < 0.001). A total of 73.78% of the respondents had a college or university education, and 61.44% were working and living in urban areas. See Table 1.

**Table 1:** BMI by socio-demographic characteristics

**Socio-demographic characteristics** **(N, %)**	**BMI Mean** **(** $\overline{\mathrm x}\pm\mathrm s$)*	**BMI < 18.5** **n(%)**	**18.5 ≤ BMI < 24** **n(%)**	**24 ≤ BMI < 28** **n(%)**^**△**^	**BMI ≥ 28** **n (%)**^★^
**Gender**					
Male (7388, 31.80)	25.50 ± 3.33	88(1.26)	2159(31.00)	3317(47.63)	1400(20.10)
Female (15,846, 68.20)	23.06 ± 3.18	826(5.64)	8708(59.46)	4086(27.90)	1024(6.99)
**Age (year)**^**a**^					
24–33 (6768, 29.13)	22.63 ± 3.60	564(9.35)	3690(61.16)	1315(21.80)	464(7.69)
34–43 (7895, 33.98)	23.88 ± 3.36	234(3.13)	3844(51.47)	2536(33.96)	854(11.44)
44–53 (6909, 29.74)	24.69 ± 3.10	96(1.46)	2727(41.56)	2859(43.57)	880(13.41)
54–63 (1405, 6.05)	24.92 ± 2.95	13(0.98)	523(39.50)	586(44.26)	202(15.26)
≥ 64 (222, 0.96)	24.60 ± 2.79	5(2.45)	73(35.78)	102(50.00)	24(11.76)
**Education**					
Junior high school or below (247, 1.06)	24.80 ± 3.19	5(2.25)	83(37.39)	105(47.30)	29(13.06)
Senior high school or secondary specialized school (5138, 22.11)	24.73 ± 3.24	100(2.07)	1947(40.38)	2047(42.45)	728(15.10)
College or undergraduate degree (17,143, 73.78)	23.58 ± 3.45	787(4.95)	8454(53.21)	5038(31.71)	1609(10.13)
Master’s degree or above (706, 3.04)	23.53 ± 3.10	22(3.25)	383(56.66)	213(31.51)	58(8.58)
**Working and living locations**					
Urban (14,276, 61.44)	23.43 ± 3.34	679(5.12)	7296(55.05)	4113(31.03)	1165(8.79)
Rural (8958, 38.56)	24.51 ± 3.44	235(2.81)	3571(42.74)	3290(39.38)	1259(15.07)
**Total**	23.85 ± 3.42	914(4.23)	10,867(50.29)	7403(34.26)	2424(11.22)

“Consent to publish” and “Not Applicable”

^a^35 missing. ^*^P < 0.05 difference between groups. ^△^Among similar groups, the overweight rate was *P* < 0.001. ^★^ Among similar groups, the obesity rate was *P* < 0.001. Underweight: BMI < 18.5 kg/m^2^; Normal: 18.5 kg/m^2^ ≤ BMI < 24.0 kg/m^2^; Overweight: 24.0 kg/m^2^ ≤ BMI < 28.0 kg/m^2^; Obese: BMI ≥ 28.0 kg/m^2 ^[24]

### Self-rated overall health status

A total of 31.19% (7247) of the respondents reported that their overall health condition was worsening, more females (31.80%) than males (29.90%) (*P* < 0.05). A total of 22.45% of the respondents reported having chronic disease(s).

### Overweight and obesity

Among the respondents 34.26% were overweight and 11.22% were obese. The mean BMI of males was 25.50 ± 3.33 (22.17–28.33), and the overweight/obesity rate was 67.73%, which was higher than the mean BMI of females (23.06 ± 3.18 (19.88–26.24)) and the overweight/obesity rate (34.89%), respectively. The overweight/obesity rates of the ≥ 64 age group (61.76%), the group with junior high school education or lower (60.36%), and rural areas (54.45%) were all higher than those of the other groups (Table 1).

**Fig. 1:**
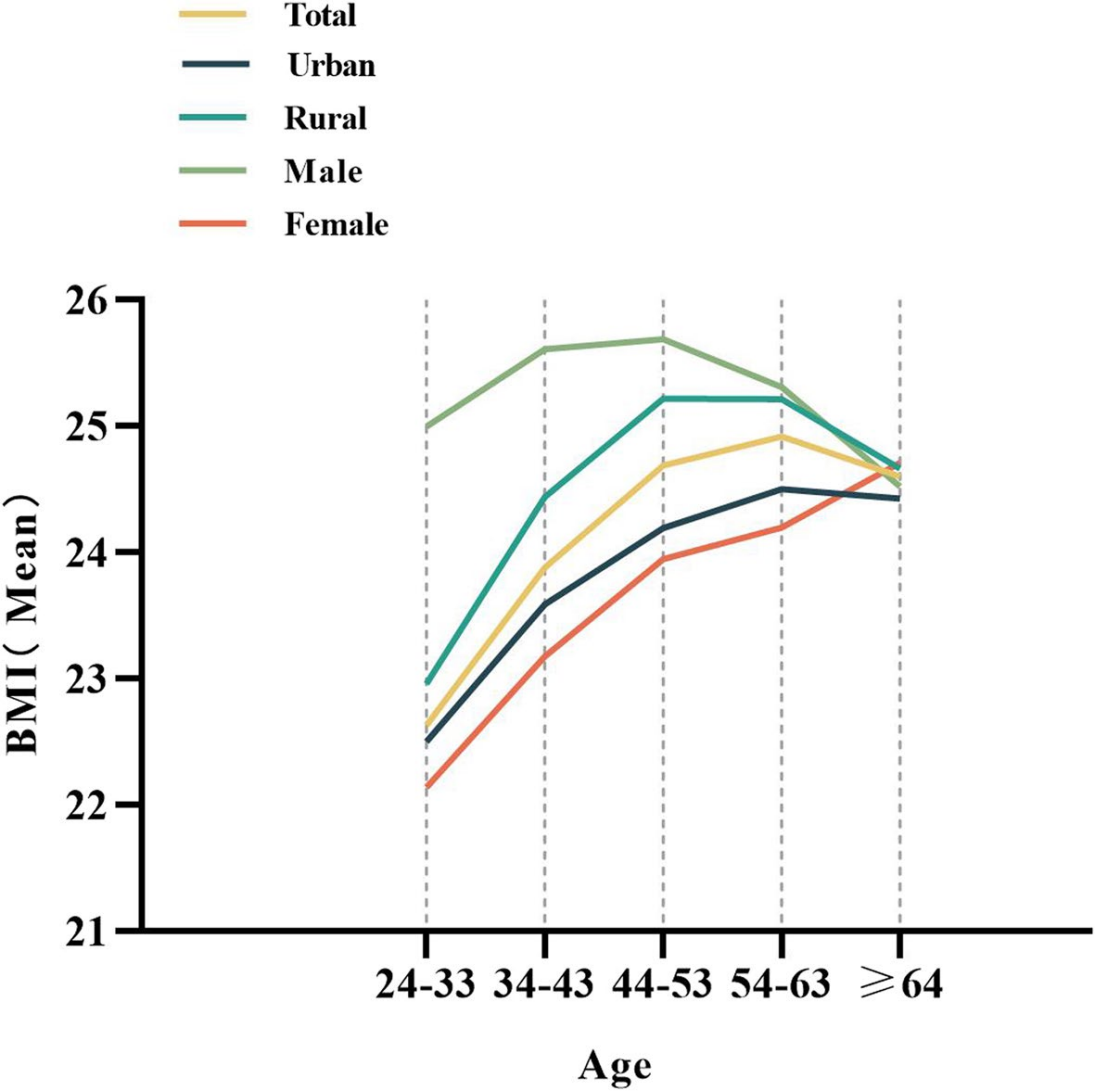
Age and BMI mean, “Consent to publish” and “Not Applicable”

**Fig. 2:**
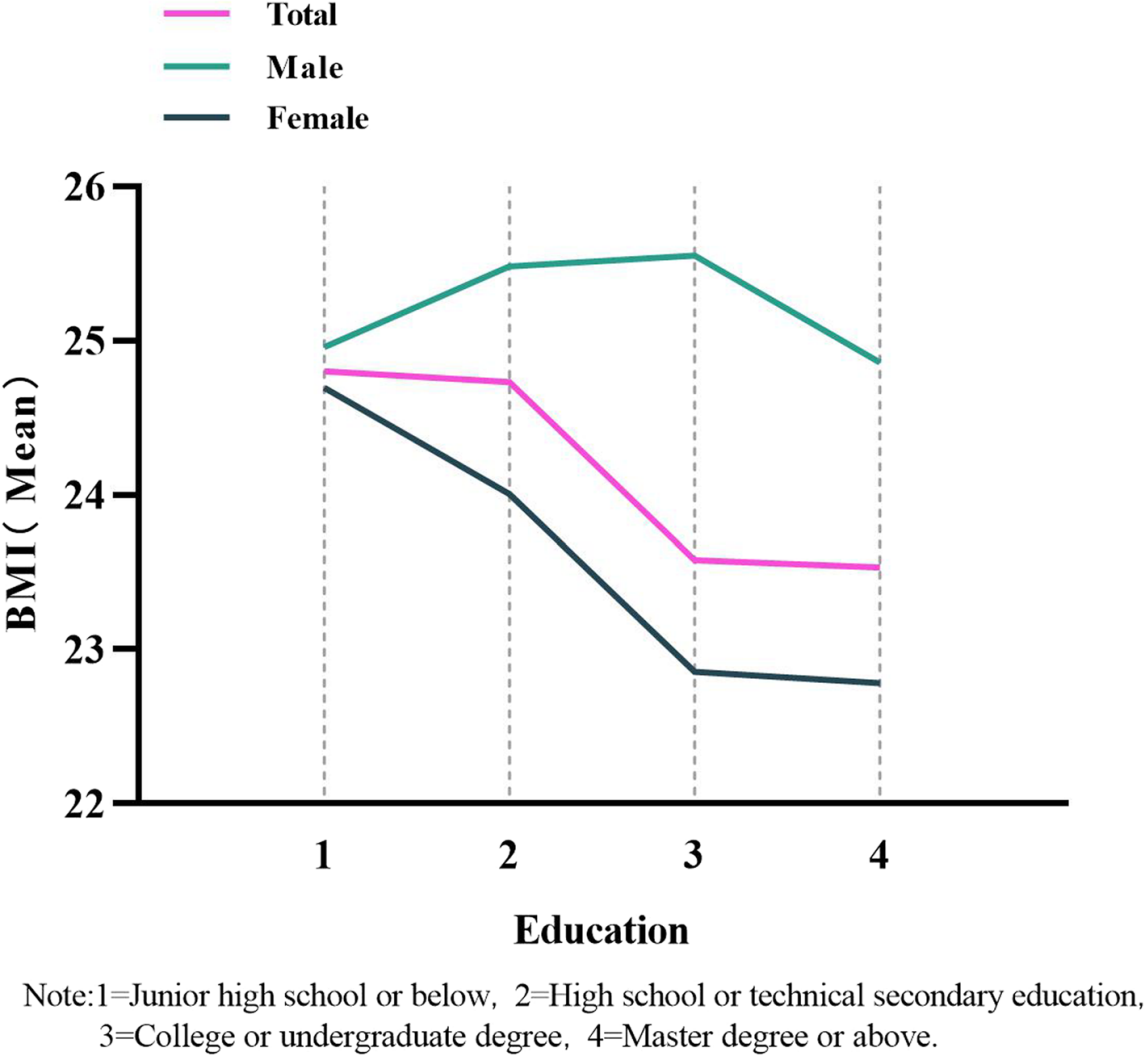
Education and BMI mean, “Consent to publish” and “Not Applicable”

**Fig. 3:**
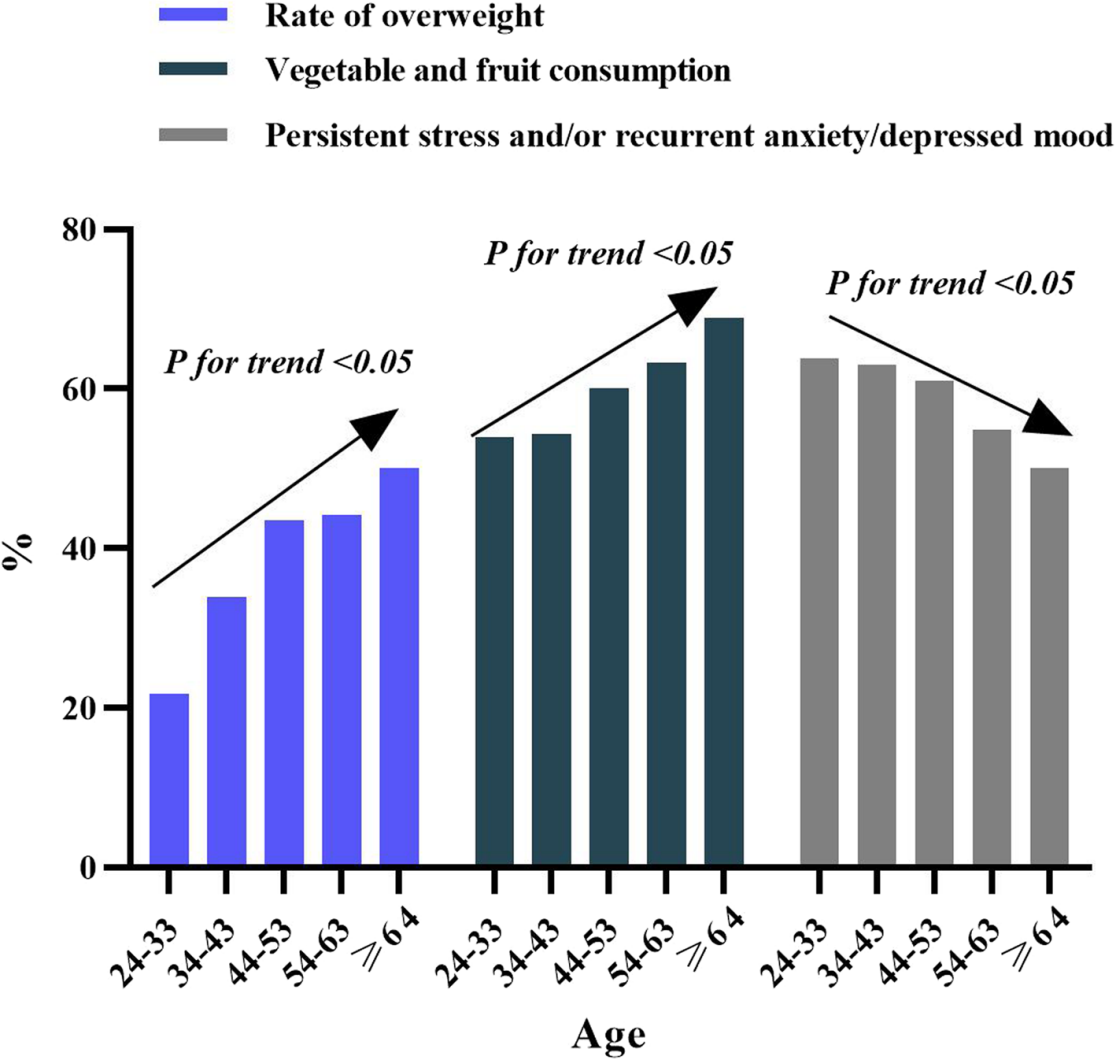
Trends in overweight rate, regularly consuming vegetables and fruits, persistent stress and/or recurrent anxiety/depressed mood and age “Consent to publish” and “Not Applicable”

The mean BMI increased with age and peaked in the 54–63 age group among both urban and rural respondents. Mean BMI increased with age, and peaked in the 44–53 age group among males (Fig. 1). The mean BMI decreased with increasing education, peaking in the group with a high school or technical secondary school education among males (Fig. 2). The rate of overweight increased with age (Fig. 3), and the trends in obesity were similar, peaking in the 54–63 age group and decreasing in the ≥ 64 age group. All of the aforementioned results were significant (*P* < 0.05).

### Lifestyle behaviors

Most of the respondents (68.17%) had regular physical activities. The proportion of males who regularly exercised (71.70%) and habitually stayed up late (67.14%) was higher than that of females (66.53% and 64.09%, respectively). Compared with other age groups, the 44–53 age group was the most physically active (75.08%), and the 34–43 age group had the highest proportion of habitually stayed up late (67.03%). Fewer urban respondents performed regular physical activities (67.09%) and habitually staying up late (64.20%) than rural respondents (69.89% and 66.42%, respectively). All of the aforementioned results were significant (*P* < 0.05) (Table 2).

**Table 2:** Lifestyle, persistent stress and/or recurrent anxiety/depressed mood of HCWs with different demographic characteristics

Variable	Regularly exercising n(%)*	Habitually staying up late n(%)*	Often eating take-out food n(%)*	Regularly consuming vegetables and fruits n(%)*	Eating breakfast regularly n(%)	Often eating fried food frequently n(%)	Often eating snack or dessert n(%)	Regularly consuming sugary drinks n(%)	Smoking n(%)*	Alcohol drinking n(%)*	Persistent stress and/or recurrent anxiety/depressed mood n(%)*
**Gender**											
Male	5297(71.70)	4960(67.14)	3088(41.80)	4284(57.99)	2654(35.92)	3255(44.06)	3074(41.61)*	2951(39.94)	1982(26.83)	2917(39.48)	4513(61.09)
Female	10,542(66.53)	10,155(64.09)	7845(49.51)	8873(56.00)	5812(36.68)	6936(43.77)	7066(44.59)*	6332(39.96)	646(4.08)	1544(9.74)	9902(62.49)
**Age (years)**^**a**^											
24–33	4153(61.36)	4490(66.34)	4121(60.89)	3653(53.97)	2959(43.72)*	2956(43.68)	3036(44.86)	2965(43.81)*	604(8.92)	1088(16.08)	4315(63.76)
34–43	5267(66.71)	5292(67.03)	4039(51.16)	4289(54.33)	2712(34.35)*	3468(43.93)	3437(43.53)	3266(41.37)*	883(11.18)	1385(17.54)	4976(63.03)
44–53	5187(75.08)	4427(64.08)	2308(33.41)	4154(60.12)	2292(33.17)*	3002(43.45)	2960(42.84)	2456(35.55)*	875(12.66)	1531(22.16)	4217(61.04)
54–63	10,450(74.38)	777(55.30)	391(27.83)	889(63.27)	409(29.11)*	659(46.90)	593(42.21)	503(35.80)*	224(15.94)	398(28.33)	771(54.88)
≥ 64	159(71.62)	100(45.05)	58(26.13)	153(68.92)	76(34.23)*	92(41.44)	100(45.05)	81(36.49)*	40(18.02)	54(24.32)	111(50.00)
**Education**											
Junior high school or lower	176(71.26)	144(58.30)	84(34.01)	164(66.40)	105(42.51)*	130(52.63)*	110(44.53)*	109(44.13)*	45(18.22)	63(25.51)	117(47.37)
High school or technical secondary education	3734(72.67)	3172(61.74)	1759(34.24)	3276(63.76)	1785(34.74)*	2406(46.83)*	2259(43.97)*	2012(39.16)*	821(15.98)	1215(23.65)	2791(54.32)
College or undergraduate degree	11,505(67.11)	11,391(66.45)	8716(50.84)	9427(54.99)	6404(37.36)*	7410(43.22)*	7520(43.87)*	6932(40.44)*	1714(10.00)	3038(17.72)	11,067(64.56)
Master’s degree or above	424(60.06)	408(57.79)	374(52.97)	290(41.08)	172(24.36)*	245(34.70)*	251(35.55)*	230(32.58)*	48(6.80)	145(20.54)	440(62.32)
**Main working and living location**											
Urban	9578(67.09)	9165(64.20)	7400(51.84)	7747(54.27)	5102(35.74)*	6098(42.72)*	6182(43.30)	5654(39.60)	1271(8.90)	2455(17.20)	9033(63.27)
Rural	6261(69.89)	5950(66.42)	3533(39.44)	5410(60.39)	3364(37.55)*	4093(45.69)*	3958(44.18)	3629(40.51)	1357(15.15)	2006(22.39)	5382(60.08)
Total	15,839(68.17)	15,115(65.06)	10,933(47.06)	13,157(56.63)	8466(36.44)	10,191(43.86)	10,140(43.64)	9283(39.95)	2628(11.31)	4461(19.20)	14,415(62.04)

“Consent to publish” and “Not Applicable”

^a^35 missing. The chi-square test was used to assess the differences between within-group and lifestyle proportions for different demographic characteristics variables. *indicates that the difference in the proportion of the same lifestyle within the demographic characteristic variable group (e.g., between genders) is statistically significant at the *P* < 0.05 level

**Fig. 4:**
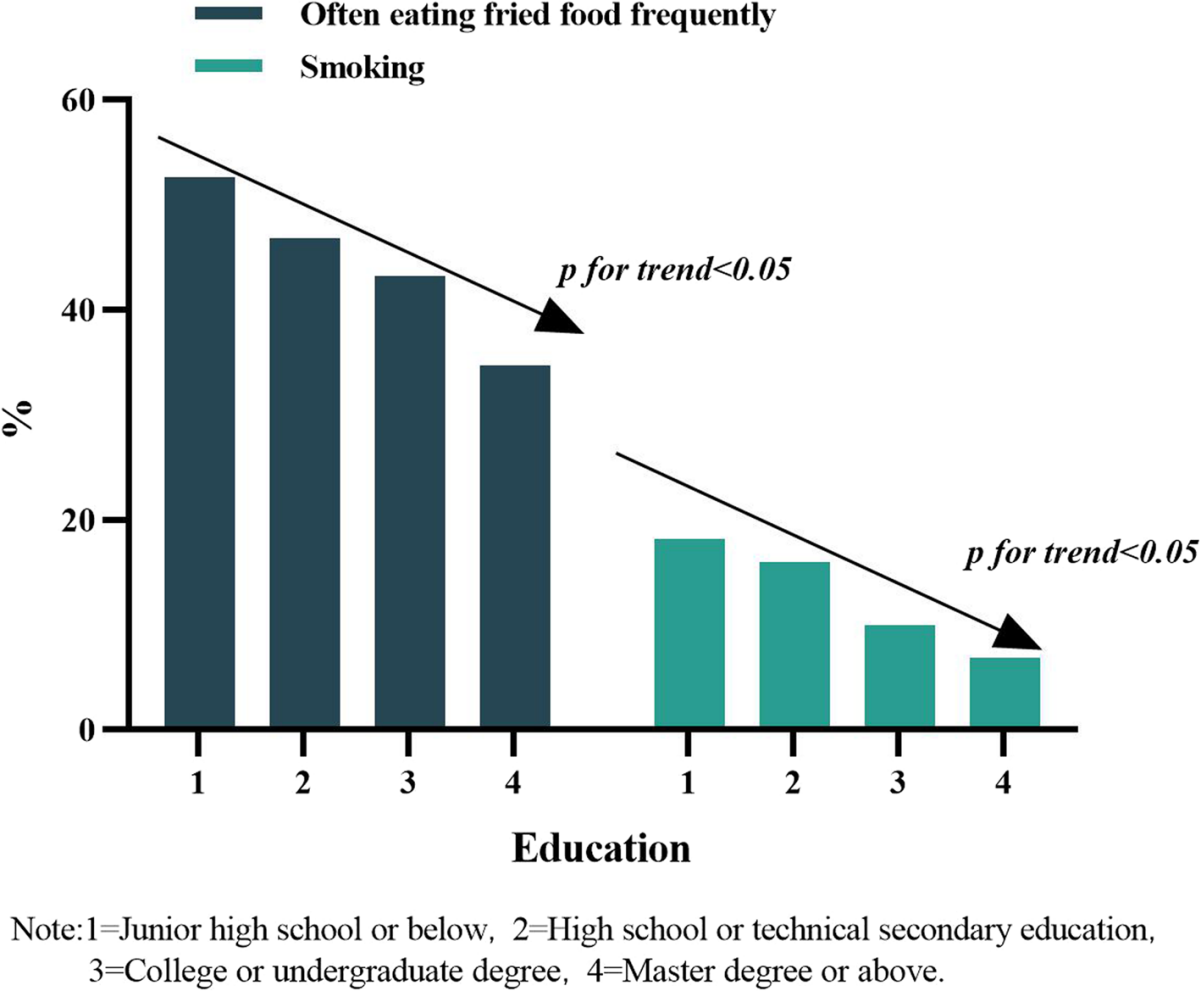
Trends of often eating fried food frequently, smoking and educational, “Consent to publish” and “Not Applicable”

The majority (56.63%) of respondents consumed vegetables and fruits regularly, which increased with age (Fig. 3). Among the education groups, those with junior high school education or lower had the highest proportion of regular consumption of vegetables and fruits (66.40%). More rural respondents consumed vegetables and fruits regularly (60.39%) than urban respondents (54.27%). A total of 43.86% of respondents often ate fried food, and the proportion decreased with increasing education level. All of the aforementioned results were significant (*P* < 0.05) (Fig. 4).

The rate of smoking was 11.31% (male: 26.83%; female: 4.08%) and decreased with increasing education level (Fig. 4). The rate of alcohol drinking was 19.20% (male: 39.48%; female: 9.74%), and more rural respondents drank alcohol (22.39%) than urban respondents (17.20%) (Table 2) (All *P* < 0.05).

As shown in Table 2, 62.04% of the respondents reported being affected by persistent stress and/or recurrent anxiety/depressed mood. The proportion of having persistent stress and/or recurrent anxiety/depressed mood among those with an education of college or undergraduate degree was 64.56%, highest among the different education groups, higher in females (62.49%) than in males (61.09%), highest (63.76%) in the 24–33 age group, and decreasing with age (Fig. 3).

### Univariate analyses of the influence of self-reported persistent stress and/or recurrent anxiety/depressed mood on lifestyle behaviors

There was a greater proportion of those with persistent stress and/or recurrent anxiety/depressed mood than among those who not habitually stayed up late (76.18% > 46.88%); often consumed take-out food (54.92% > 34.21%), fried food (49.93% > 33.94%), snacks or desserts (50.51% > 32.42%) and sugary drinks (46.57% > 29.14%); smoked (14.27% > 6.47%) and consumed alcohol (23.34% > 12.43%). All of the aforementioned results were significant (*P* < 0.001) (Table 3).

**Table 3:** Univariate analyses of the influence of persistent stress and/or recurrent anxiety/depressed mood on lifestyle behaviors

Lifestyle behaviors	Persistent stress and/or recurrent anxiety/depressed mood		*P*
	Yes	No	
**Regularly exercising**	10,323(71.61)	5516(62.55)	** < 0.001**
**Habitually staying up late**	10,981(76.18)	4134(46.88)	** < 0.001**
**Regularly consuming vegetables and fruits**	7916(54.92)	3017(34.21)	** < 0.001**
**Often eating take-out food**	8568(59.44)	4589(52.04)	** < 0.001**
**Eating breakfast regularly**	6188(42.93)	2278(25.83)	** < 0.001**
**Often eating fried food**	7198(49.93)	2993(33.94)	** < 0.001**
**Often eating snacks or desserts**	7281(50.51)	2859(32.42)	** < 0.001**
**Regularly consuming sugary drinks**	6713(46.57)	2570(29.14)	** < 0.001**
**Smoking**	2057(14.27)	571(6.47)	** < 0.001**
**Alcohol drinking**	3365(23.34)	1096(12.43)	** < 0.001**

“Consent to publish” and “Not Applicable”

### Multivariate binary logistic regression analysis of risk factors for overweight/obesity

Logistic regression analysis showed that gender (female) (OR: 0.314, 95% CI: 0.292–0.336), age (OR: 1.742–2.334, 95% CI: 1.544–2.858), and education (OR: 0.620–0.728, 95% CI: 0.445–0.973), working and living area (urban/rural) (OR: 1.271, 95% CI: 1.192–1.355), and consumption of breakfast (OR: 0.898, 95% CI: 0.839–0.960), fried food (OR: 1.133, 95% CI: 1.048–1.224), and alcohol drinking (OR: 1.111, 95% CI: 1.017–1.214) were factors for overweight/obesity. All of the aforementioned results were significant (*P* < 0.05) (Table 4).

**Table 4:** Multi-variate binary logistic regression analysis of influencing factors of overweight/obesity in HCWs

Variables	OR	*95%CI*		*P*
		**Low**	**High**	
**Gender (Reference: male)**	0.314	0.292	0.336	** < 0.001**
**Age (yrs) **^**a**^** (Reference: 24–33 yrs)**				
34–43	1.742	1.616	1.878	** < 0.001**
44–53	2.334	2.149	2.535	** < 0.001**
54–63	1.894	1.650	2.172	** < 0.001**
≥ 64	2.101	1.544	2.858	** < 0.001**
**Degree of education (Reference: junior high school or lower)**				
High school or technical secondary education	0.796	0.595	1.064	0.124
College or undergraduate degree	0.728	0.545	0.973	**0.032**
Master’s degree or above	0.620	0.445	0.865	**0.005**
**Main working and living location (Reference: urban as)**	1.271	1.192	1.355	** < 0.001**
**Regularly exercising (Reference: No)**	1.003	0.939	1.072	0.922
**Habitually staying up late (Reference: No)**	1.054	0.987	1.127	0.118
**Often eating take-out food (Reference: No)**	1.032	0.967	1.101	0.347
**Regularly consuming vegetables and fruits (Reference: No)**	0.956	0.894	1.021	0.178
**Eating breakfast regularly (Reference: No)**	0.898	0.839	0.960	**0.002**
**Often eating fried food frequently (Reference: No)**	1.133	1.048	1.224	**0.002**
**Often eating snack or dessert (Reference: No)**	1.000	0.92	1.087	0.999
**Regularly consuming sugary drinks** **(Reference: No)**	1.061	0.979	1.151	0.148
**Smoking (Reference: No)**	0.949	0.851	1.059	0.349
**Alcohol drinking (Reference: No)**	1.111	1.017	1.214	**0.02**
**persistent stress and/or recurrent anxiety/depressed mood (Reference: No)**	0.972	0.912	1.036	0.381

^a^35 missing

“Consent to publish” and “Not Applicable”

## Discussion

Overweight/obesity is an important factor affecting the health and normal work of HCWs. This study randomly surveyed HCWs across China on overweight/obesity, self-rated overall health status, persistent stress and/or recurrent anxiety/depressed mood and related lifestyle behaviors, and analysed the risk factors for overweight/obesity. This study found that the overweight/obesity rate of HCWs was 45.48%, which was lower than that of the overall population of China (49.6%) [28].

Contrary to the findings of other studies with no significant difference in obesity rates between male and female HCWs, [29] this study showed that the mean BMI, and rates of overweight and obesity in male HCWs were significantly higher than those of females. Logistic regression analysis showed that the risk of overweight/obesity in females was approximately one third of that in males. Higher male overweight/obesity might be related to more lifestyle risk factors. This survey showed that the proportion of men who habitually stayed up late, smoked and drank alcohol was significantly higher than that of women.

China Health and Nutrition Survey (1991–2015) has revealed an inverted U-shaped relationship between overweight/obesity and age, peaking at an approximate age of 50 years [30]. The current study revealed a similar pattern, with both BMI values and obesity rates peaking in the 54–63 age group (Table 2). Logistic regression analyses showed that after controlling for other confounding factors, the risk of overweight/obesity in all age groups was higher than that in the 24–33 year age group, and the risk of overweight/obesity in the 44–53 year age group was the highest, which was 2.33 times that in the 24–33 year age group, which might be related to the decrease in regular exercise with ageing. According to the 2020 National Fitness Survey Communique released by the National Physical Monitoring Center, the proportion of people who took part in physical exercise once a week or more showed a declining trend with age [31]. This study showed that the relationship between BMI and age of male HCWs was consistent with the results of China Health and Nutrition Survey (1991–2015), [30] which peaked in the 44–53 and 54–63 age groups, respectively. However, the BMI of female HCWs increased with age, which might be related to the mood change resulting from estrogen decrease or menopause [32].

A study showed that the prevalence of obesity was lower in better educated people than in those with less education, [33] and the present study found a similar phenomenon. Compared with those with junior high school education level or below, HCWs with higher education level were less likely to be overweight or obese. Unhealthy lifestyles might be a mediator between low education level and overweight/obesity [34].

This study demonstrated that both the mean BMI and overweight/obesity rate of HCWs living and working in rural areas were higher than those of urban HCWs, which may be related to more unhealthy lifestyles among rural HCWs. This study showed that the proportions of habitually staying up late, often eating fried food and drinking alcohol were higher in rural than in urban HCWs. Although rural HCWs were better than their urban counterparts in terms of lifestyle behaviors such as regular physical exercise, consumption of vegetables and fruits, and eating breakfast, this may not be enough to neutralize the effect of rurality on weight gain. Logistic regression analyses showed that when other factors were controlled for, the risk factors for overweight and obesity were frequent consumption of fried food and alcohol.

This study found that 22.45% of HCWs reported having chronic diseases, which was significantly lower than the national average (34.29%) [35]. This survey showed that the overall alcohol drinking rate of HCWs was 19.20%, which was lower than that of Chinese adults in general (43.7%) [36]. The smoking rate of HCWs was 11.31%, which was significantly lower than that of common adults across China (26.6%); [37] however, the smoking rate in this study was significantly higher than that in a previous study among HCWs [38]. The regular exercise rate was 68.17%, which was significantly higher than that of common Chinese adults [31]. The increase in smoking rate might be related to the sudden increase in work stress caused by the COVID-19 pandemic. Petrelli et al. found that HCWs may use smoking as a behavioral option to cope with excessive work pressure [39].

Logistic regression analyses showed that those who frequently skipped breakfast were more likely to be overweight or obese, which was consistent with the findings of a review [40]. It has been confirmed that breakfast skipping can lead to more daily energy intake and is more likely to result in weight gain. The analyses found that those who often ate fried food were more likely to be overweight/obese, which was consistent with the findings of Kriaucioniene V. et al. [41]. Additionally, the results showed that alcohol drinkers were more likely to be overweight/obese than those who did not drink alcohol, thus indicating the effects of alcohol consumption on weight gain [42].

This survey found that HCWs with persistent stress and/or recurrent anxiety/depressed mood were more likely to stay up late habitually, often consume take-out food, fried food, snacks or desserts, sugary drinks, smoke cigarettes and drink alcohol than those without persistent stress and/or recurrent anxiety/depressed mood. This is consistent with other research results [29, 43]. This survey also found that overweight/obesity was statistically significantly associated with persistent stress and/or recurrent anxiety/depressed mood for the first time among Chinese HCWs when lifestyles were controlled as confounders. The probable mechanism might be that the extra stress of Chinese HCWs due to the pandemic and the intensive work pattern activated the hypothalamic‒pituitary‒adrenal axis (HPA), promoting cortisol secretion and resulting in abdominal body fat accumulation and intake of "comfort food" [10]. Another possible mechanism might be that persistent stress and/or recurrent anxiety/depressed mood play a substantial role in sleeping disorders and physical inactivity [44, 45].

The persistent stress and/or recurrent anxiety/depressed mood of Chinese HCWs stem from various aspects. First, many clinical health care workers are overworked [46]. For example, in 2021 alone, the number of outpatient visits to Chinese medical institutions reached 8.47 billion [47]. Second, they fully participated in the prevention, control and treatment of COVID-19, were repeatedly isolated for a long periods, and faced the risk of being infected at any time, which made them suffer from additional pressure. Third, there are few psychological counselling and stress relief services for HCWs, making it difficult to obtain timely and effective psychological support. These factors also seriously affected the lifestyles of HCWs, making it difficult for them to adhere to a balanced diet, regular routines, and physical exercise.

To fundamentally address the problem of obesity and overweight among HCWs, at the national level, the right to rest and physical and mental health promotion should be legally ensured [48, 49]. At the institutional level, a system of rotational rest for HCWs should be established, especially in the event of a pandemic, while ensuring institutional continuity. Second, regular psychological counselling and support services should be provided to ensure that their stress and emotions are relieved in a timely manner [50, 51]. Third, routine healthy lifestyle guidance (e.g., by health coaches) and modification of unhealthy behaviors (e.g., alcohol addiction, sedentary habits, and high calorie intake) should be provided systematically and specifically [52]. Fourth, for those who suffer from obesity or have serious psychological and emotional problems, therapeutic intervention services should be given based on the principle of informed voluntary participation [53].

This study might have the following limitations. The data in this study were collected by way of self-reporting through an online survey, and the accuracy of the data largely depended on the ability or willingness of the respondents to comprehend and answer the questions; therefore, the findings may be underestimated or exaggerated. Hence, these conclusions should be viewed cautiously. A study has shown that nurses have a higher risk of obesity than other occupational categories among HCWs, [54] but this study did not classify the occupations of HCWs and perform further analyses. However, given that the sample size of this study is large enough to compensate for the above shortcomings to some extent, the conclusions are still acceptable.

## Conclusions

The overweight/obesity rate of Chinese HCWs is rather high, and more than half of them have undergone excessive persistent stress and/or recurrent anxiety/depressed mood. Overweight/obesity of HCWs is directly associated with lifestyle behaviors such as increasing consumption of fried food and alcohol drinking. However, it is probably the case that the unhealthy lifestyle behaviors originated from persistent stress and/or recurrent anxiety/depressed mood. Stress reduction measures should therefore be implemented for the control of overweight/obesity among HCWs.

## Data Availability

The datasets used and/or analysed during the current study are available from the corresponding author on reasonable request.

## Abbreviations

HCWs: Healthcare workers
HPA: Hypothalamic–pituitary–adrenal axis
DALYs: Disability-adjusted life years
